# Expected Utility Based Decision Making under *Z*-Information and Its Application

**DOI:** 10.1155/2015/364512

**Published:** 2015-08-03

**Authors:** Rashad R. Aliev, Derar Atallah Talal Mraiziq, Oleg H. Huseynov

**Affiliations:** ^1^Department of Mathematics, Eastern Mediterranean University, Famagusta, Northern Cyprus, Mersin 10, Turkey; ^2^Department of Computer-Aided Control Systems, Azerbaijan State Oil Academy, 20 Azadlig Avenue, 1010 Baku, Azerbaijan

## Abstract

Real-world decision relevant information is often partially reliable. The reasons are partial reliability of the source of information, misperceptions, psychological biases, incompetence, and so forth. *Z*-numbers based formalization of information (*Z*-information) represents a natural language (NL) based value of a variable of interest in line with the related NL based reliability. What is important is that *Z*-information not only is the most general representation of real-world imperfect information but also has the highest descriptive power from human perception point of view as compared to fuzzy number. In this study, we present an approach to decision making under *Z*-information based on direct computation over *Z*-numbers. This approach utilizes expected utility paradigm and is applied to a benchmark decision problem in the field of economics.

## 1. Introduction

Decision making is one of the attractive research areas in the last decades. The complexity and uncertainty are persistent phenomenon in the real world, and the fuzzy set [1–3] is widely used in decision making process [3, 4]. Much of decision based information is uncertain. Human has a high capability of making logical decisions based on uncertain, incomplete, and/or inaccurate information [5].

*Z*-number is a sufficient formalization of real-world information that should roughly be considered in light of its reliability. The critical issue is that the reliability of information is not considered properly. Zadeh has proposed a new notion *Z*-number which is more appropriate to describe the uncertainty. *Z*-number takes both restraint and reliability. In comparison with the classical fuzzy number, *Z*-number has more ability to describe the real information of human [6].

*Z*-numbers were firstly presented by Zadeh in 2011 [5], and afterwards the researchers started to discuss *Z*-numbers in decision making under uncertainty and in many other fields. One of the main goals of *Z*-number is to produce fuzzy numbers with degree of self-confidence in order to know the real information. By using the *Z*-number the knowledge of human can be represented in a better way [7].

The computations with *Z*-numbers can be viewed as a generalization of computations with numbers, intervals, fuzzy numbers, and random numbers. As specified, the levels of generality can be separated as follows: computation with numbers (ground level zero); computation with intervals (level one); computation with fuzzy numbers (level two) [2]; computation with random numbers (level two); and computation with *Z*-numbers (level three). The capability of building realistic models of real-world systems is increased by the increase of the generality level, especially in the realms of economics, risk assessment, decision analysis, planning, and analysis of causality [5].

In [4] the authors suggest an approach to use *Z*-numbers for solving multicriteria decision making problem. For computation over *Z*-numbers some operations are suggested that are based on Zadeh's extension principle [5]. *Z*-numbers are also used for the purpose of reasoning [8]. In [6] proposed approach is intended to use *Z*-numbers for the expected utility application to solve decision making problems. An approach to use *Z*-numbers for answering questions and decisions making is considered in [9]. *Z*-numbers converted into classical fuzzy numbers are suggested in [4, 9]. In [7], *Z*-numbers are converted into classical fuzzy numbers and the fuzzy numbers are converted into crisp numbers. In [10] the theoretical approach for computing arithmetic operations over discrete *Z*-numbers is proposed.

In [11] authors suggest general and computationally effective theoretic approach to computations with discrete *Z*-numbers. The authors provide strong motivation of the use of discrete *Z*-numbers as an alternative to the continuous counterparts. In particular, the motivation is based on the fact that NL based information has a discrete framework. The suggested arithmetic of *Z*-numbers includes basic arithmetic operations and important algebraic operations over *Z*-numbers. The proposed approach allows dealing with *Z*-information directly.

This paper focuses on investigating an approach for decision making which generalizes the expected utility approach of *Z*-information. This approach is based on direct computation over *Z*-numbers without converting them to fuzzy numbers and differed from the existing works used for decision making problems. The direct computation of *Z*-numbers without conversion eliminates the loss of information. In this research we recommend an approach based on expected utility to solve the decision making problems with *Z*-information. This approach is based on computation over *Z*-numbers according to operations suggested in [5, 10]. At the end, we provide a numerical example of the proposed approach to solve a benchmark problem.

This paper is organized as follows. The preliminaries for *Z*-numbers are reviewed in Section 2.  Section 3 describes the numerical computations with discrete *Z*-numbers.  Section 4 is devoted to statement and solution of a considered decision problem with *Z*-information.  Section 5 consists of application, and the conclusions are revealed in Section 6.

## 2. Preliminaries

**Definition 1 (a discrete fuzzy number [12–14]).**

A fuzzy subset *A* of the real line *ℛ* with membership function *μ*_*A*_ : *ℛ* → [0,1] is a discrete fuzzy number if its support is finite; that is, there exist *x*_1_,…, *x*_*n*_ ∈ *ℛ* with *x*_1_ < *x*_2_ < ⋯<*x*_*n*_, such that supp⁡(*A*) = {*x*_1_,…, *x*_*n*_} and there exist natural numbers *s*, *t* with 1 ≤ *s* ≤ *t* ≤ *n* satisfying the following conditions:

(1) *μ*_*A*_(*x*_*i*_) = 1 for any natural number *i* with *s* ≤ *i* ≤ *t*;
(2) *μ*_*A*_(*x*_*i*_) ≤ *μ*_*A*_(*x*_*j*_) for any natural numbers *i*, *j* with 1 ≤ *i* ≤ *j* ≤ *s*;
(3) *μ*_*A*_(*x*_*i*_) ≥ *μ*_*A*_(*x*_*j*_) for any natural numbers *i*, *j* with *t* ≤ *i* ≤ *j* ≤ *n*.

**Definition 2 (probability measure of a discrete fuzzy number [15]).**

Let *A* be a discrete fuzzy number. A probability measure of *A* denoted by *P*(*A*) is defined as

(1) PA=∑i=1nμAxipxi=μA(xj1)pj(xj1)+μA(xj2)pj(xj2) +⋯+μAxjnjpjxjnj.

Below we present the definition of addition of discrete fuzzy numbers suggested in [12–14, 16], where noninteractive fuzzy numbers are considered.

**Definition 3 (addition of discrete fuzzy numbers [12–14, 16]).**

The addition of discrete fuzzy numbers ${\tilde{A}}_{12}={\tilde{A}}_{1}+{\tilde{A}}_{2}$ is a discrete fuzzy number whose *α*-cut is given as [12–14, 16]

(2) A12α=x∈supp⁡(A~1)+supp⁡(A~2) ∣     min⁡{A1α+A2α}≤x≤max⁡{A1α+A2α}(A~1),

where

(3) supp⁡(A~1)+supp⁡(A~2) =x1+x2 ∣ xj∈supp⁡(A~j), j=1,2min⁡{A~1α+A~2α}=min⁡{x1+x2 ∣ xj∈A~jα, j=1,2}max⁡{A~1α+A~2α}=max⁡{x1+x2 ∣ xj∈A~jα, j=1,2}μA~1+A~2x=sup⁡α∈0,1x∈A~1α+A~2α.

**Definition 4 (multiplication of discrete fuzzy numbers [10, 11]).**

The multiplication of discrete fuzzy numbers ${\tilde{A}}_{12}={\tilde{A}}_{1}\cdot {\tilde{A}}_{2}$ is a discrete fuzzy number whose *α*-cut is given as [10]

(4) A12α=x∈{supp⁡(A~1)·supp⁡(A~2)} ∣   min⁡{A1α·A2α}≤x≤max⁡{A1α·A2α}(A~1),

where

(5) supp⁡A~1·supp⁡A~2  ={x1·x2 ∣ xj∈supp⁡(A~j), j=1,2}min⁡{A~1α·A~2α}=min⁡{x1·x2 ∣ xj∈A~jα, j=1,2}max⁡{A~1α·A~2α}=max⁡{x1·x2 ∣ xj∈A~jα, j=1,2}μA~1·A~2x=sup⁡α∈0,1x∈A~1α·A~2α.

**Definition 5 (discrete probability distribution).**

The discrete probability distribution is defined as a function *p* where if we suppose a discrete random variable *X* taking *K* different values with probability that *X* = *x*_*i*_ defined to be *P*(*X* = *x*_*i*_) = *p*(*x*_*i*_), the probability *p*(*x*_*i*_) must satisfy 0 ≤ *p*(*x*_*i*_) ≤ 1 for each *i* and ∑_*i*=1_^*k*^ *p*(*x*_*i*_) = 1 [17].

**Definition 6 (convolution of discrete probability distributions).**

Suppose *X*_1_ and *X*_2_ are two discrete random variables with distribution functions *p*_1_ and *p*_2_. The distribution function *X*_1_∗*X*_2_ is given as [17]

(6) $$\begin{array}{c} p_{12}\left(x\right)=\underset{x=x_{1}*x_{2}}{\sum }p_{1}\left(x_{1}\right)p_{2}\left(x_{2}\right). \end{array}$$

**Definition 7 (a discrete *Z*-number [11]).**

A discrete *Z*-number is defined as an ordered pair $Z=(\tilde{A},\tilde{B})$, where $\tilde{A}$ and $\tilde{B}$ are discrete fuzzy numbers, $\tilde{A}$ is a fuzzy constraint on values that a random variable *X* may take, and $\tilde{B}$ which has a membership function $\mu_{\tilde{A}}$ is a fuzzy constraint on the probability measure of $\tilde{A}$:

(7) $$\begin{array}{c} P\left(\tilde{A}\right)\;\;\text{is}\;\;\tilde{B}. \end{array}$$

The concept of a restriction has more generality than the concept of a constraint [18]. A restriction may be observed as a generalized constraint. A probability distribution is a restriction but is not a constraint [19].

*Z* ^+^-number concept is related to discrete *Z*-number; that is, *Z* ^+^-number is a pair of fuzzy number $\tilde{A}$ and random number *R* to be defined as

(8) $$\begin{array}{c} Z^{+}=\left(\tilde{A},R\right), \end{array}$$

where $\tilde{A}$ plays the same role as in discrete *Z*-number $Z=(\tilde{A},\tilde{B})$, and *R* plays the role of the probability distribution *P* such that [10]

(9) PA=∑i=1nμAxipxi=μA(xj1)pj(xj1)+μA(xj2)pj(xj2) +⋯+μAxjnjpjxjnj.

## 3. Computation with Discrete *Z*-Numbers

### 3.1. General Review

Zadeh has suggested a general approach for computations with *Z*-numbers according to Zadeh's extension principle [5]. This study is very complex in comparison with the previous one. The researchers look into using *Z*-numbers, but the lack of a direct and easy way to compute *Z*-numbers forced them to start thinking about a way to convert them into fuzzy numbers.

In [9] authors suggest an approach to convert *Z*-numbers into classical fuzzy numbers. They convert the second part to crisp number, but this leads to loss of original information.

The studies [4, 7, 20] are used according to what has been put forward in the study [9], but in fact this method does not give the results of high reliability. Therefore, the researchers looked for a new and simple way to calculate *Z*-numbers directly without conversion, based on what has been suggested in the study [5].

### 3.2. Addition and Multiplication of Discrete *Z*-Numbers

Assume $Z_{1}=({\tilde{A}}_{1},{\tilde{B}}_{1})$ and $Z_{2}=({\tilde{A}}_{2},{\tilde{B}}_{2})$ be discrete *Z*-numbers describing values of uncertain real valued variables *X*_1_ and *X*_2_. The addition and multiplication of *Z*-numbers *Z*_12_ = *Z*_1_∗*Z*_2_, ∗ ∈ {+, ×} are determined as follows [11]. Let $Z_{1}^{+}=({\tilde{A}}_{1},R_{1})$ and $Z_{2}^{+}=({\tilde{A}}_{2},R_{2})$ be given. Then

(10) $$\begin{array}{c} Z_{12}^{+}=Z_{1}^{+}*Z_{2}^{+}=\left({\tilde{A}}_{1}*{\tilde{A}}_{2},R_{1}*R_{2}\right), \end{array}$$

where *R*_1_ and *R*_2_ are represented by discrete probability distributions (Definition 5):

(11) $$\begin{array}{c} p_{1}=p_{1}\left(x_{11}\right)\setminus x_{11}+p_{1}\left(x_{12}\right)\setminus x_{12}+\cdots +p_{1}\left(x_{1n}\right)\setminus x_{1n}\\ p_{2}=p_{2}\left(x_{21}\right)\setminus x_{21}+p_{2}\left(x_{22}\right)\setminus x_{22}+\cdots +p_{2}\left(x_{2n}\right)\setminus x_{2n}. \end{array}$$

${\tilde{A}}_{12}={\tilde{A}}_{1}*{\tilde{A}}_{2}$ is a sum (or multiplication) of fuzzy numbers defined on the basis of Definition 3 (Definition 4) and *R*_1_∗*R*_2_ is a convolution of probability distribution defined on the basis of Definition 6.

Next, we should construct ${\tilde{B}}_{12}$ by solving the following problem:

(12) $$\begin{array}{c} \mu_{{\tilde{B}}_{12}}\left(b_{12s}\right)=\sup\left(\mu_{p_{12s}}\left(p_{12s}\right)\right) \end{array}$$

subject to

(13) $$\begin{array}{c} b_{12s}=\underset{i}{\sum }p_{12s}\left(x_{i}\right)\mu_{{\tilde{A}}_{12}}\left(x_{i}\right),\\ \mu_{p_{12}}\left(p_{12}\right)=\underset{\left\{p_{1},p_{2}\;|\;p_{12}=p_{1}\circ p_{2}\right\}}{\max}\left[\mu_{p_{1}}\left(p_{1}\right)\wedge \mu_{p_{2}}\left(p_{2}\right)\right],\\ \mu_{p_{j}}\left(p_{j}\right)=\mu_{{\tilde{B}}_{j}}\left(\overset{n}{\underset{k=1}{\sum }}\mu_{{\tilde{A}}_{j}}\left(u_{k}\right)p_{j}\left(u_{k}\right)\right),\;j=1,2. \end{array}$$

Thus, *Z*_12_ = *Z*_1_∗*Z*_2_ is obtained as $Z_{12}=({\tilde{A}}_{12},{\tilde{B}}_{12})$ [10, 11].

### 3.3. Ranking of Discrete *Z*-Numbers [11]

**Figure 1:**
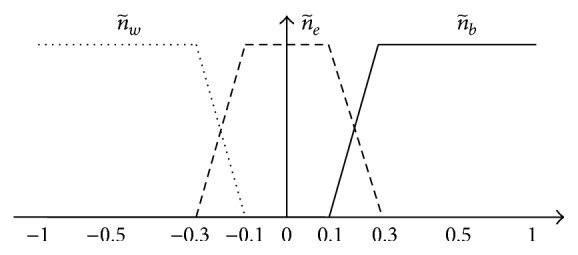
The membership functions of ${\tilde{n}}_{b}$, ${\tilde{n}}_{e}$, ${\tilde{n}}_{w}$.

Ranking of discrete *Z*-numbers is a necessary operation in arithmetic of *Z*-numbers and is a challenging practical issue. Zadeh addresses the problem of ranking *Z*-numbers as a very important problem [5]. In contrast to real numbers, *Z*-numbers are ordered pairs, for ranking of which there can be no unique approach. We suggest considering comparison of *Z*-numbers on the basis of fuzzy optimality (FO) principle. Let *Z*-numbers $Z_{1}=({\tilde{A}}_{1},{\tilde{B}}_{1})$ and $Z_{2}=({\tilde{A}}_{2},{\tilde{B}}_{2})$ be given. First, it is needed to calculate the functions *n*_*b*_, *n*_*e*_, *n*_*w*_ which evaluate how much one of the *Z*-numbers is better, equivalent, and worse than the other one with respect to the first and the second components [11]:

(14) $$\begin{array}{c} n_{b}\left(Z_{i},Z_{j}\right)=Ps_{b}\left({\tilde{\delta }}_{\tilde{A}}^{i,j}\right)+Ps_{b}\left({\tilde{\delta }}_{\tilde{B}}^{i,j}\right),\\ n_{e}\left(Z_{i},Z_{j}\right)=Ps_{e}\left({\tilde{\delta }}_{\tilde{A}}^{i,j}\right)+Ps_{e}\left({\tilde{\delta }}_{\tilde{B}}^{i,j}\right),\\ n_{w}\left(Z_{i},Z_{j}\right)=Ps_{w}\left({\tilde{\delta }}_{\tilde{A}}^{i,j}\right)+Ps_{w}\left({\tilde{\delta }}_{\tilde{B}}^{i,j}\right), \end{array}$$

where ${\tilde{\delta }}_{\tilde{A}}^{i,j}={\tilde{A}}_{i}-{\tilde{A}}_{j}$, ${\tilde{\delta }}_{\tilde{B}}^{i,j}={\tilde{B}}_{i}-{\tilde{B}}_{j}$:

(15) Pslδ~A~i,j=Possδ~A~i,j ∣ nl∑t∈b,e,wPossδ~A~i,j ∣ nt,Pslδ~B~i,j=Possδ~B~i,j ∣ nl∑t∈b,e,wPossδ~B~i,j ∣ nt,t∈b,e,w,

where *i*, *j* = 1,2, *i* ≠ *j*. As $\sum_{t\in \{b,e,w\}}^{}Ps_{l}({\tilde{\delta }}_{k}^{i,j})=1$ always holds, one always has *n*_*b*_(*Z*_*i*_, *Z*_*j*_) + *n*_*e*_(*Z*_*i*_, *Z*_*j*_) + *n*_*w*_(*Z*_*i*_, *Z*_*j*_) = *N*, where *N* is the number of components of a *Z*-number; that is, *N* = 2. The membership functions of ${\tilde{n}}_{b}$, ${\tilde{n}}_{e}$, ${\tilde{n}}_{w}$ are shown in Figure 1 [11].

Next it is needed to determine the greatest *k* such that *Z*_*i*_ Pareto dominates *Z*_*j*_ to the degree (1 − *k*). For this purpose, a function *d* is introduced:

(16) dZi,Zj =0,if  nbZi,Zj≤2−neZi,Zj2,2·nb(Zi,Zj) + ne(Zi,Zj)−2 ×nbZi,Zj−1,otherwise.

Given *d*, the desired greatest *k* is found as *k* = 1 − *d*(*Z*_*i*_, *Z*_*j*_), and then (1 − *k*) = *d*(*Z*_*i*_, *Z*_*j*_). *d*(*Z*_*i*_, *Z*_*j*_) = 1 implies Pareto dominance of *Z*_*i*_ over *Z*_*j*_, whereas *d*(*Z*_*i*_, *Z*_*j*_) = 0 implies no Pareto dominance of *Z*_*i*_ over *Z*_*j*_. The degree of optimality do(*Z*_*i*_) is determined as follows:

(17) $$\begin{array}{c} \text{do}\left(Z_{i}\right)=1-d\left(Z_{j},Z_{i}\right). \end{array}$$

Thus, in other words, do(*Z*_*i*_) is the degree to which one *Z*-number is higher than the other one. Then [11]

(18) $$\begin{array}{c} Z_{i}>Z_{j}\;\text{if}\;\;\text{and}\;\;\text{only}\;\;\text{if}\;\;\text{do}\left(Z_{i}\right)>\text{do}\left(Z_{j}\right),\\ Z_{i}<Z_{j}\;\text{if}\;\;\text{and}\;\;\text{only}\;\;\text{if}\;\;\text{do}\left(Z_{i}\right)<\text{do}\left(Z_{j}\right),\\ \text{do}\left(Z_{i}\right)=\text{do}\left(Z_{j}\right),\;\text{otherwise}. \end{array}$$

Recall that comparison of fuzzy numbers is also a matter of a degree due to related vagueness. For *Z*-numbers, which are more complex constructs characterized by possibilistic-probabilistic uncertainty, degree-based comparison is even more desirable.

The suggested approach may be considered as basis of a human-oriented ranking of *Z*-numbers. In this viewpoint, we suggest taking into account the degree of pessimism *β* ∈ [0,1] as a mental factor which influences a choice of a preferred *Z*-number. The degree of pessimism is submitted by a human observer who wishes to compare the considered *Z*-numbers but does not completely rely on the results obtained by the above mentioned fuzzy optimality approach. In this viewpoint, given do(*Z*_*j*_) ≤ do(*Z*_*i*_), we define for two *Z*-numbers *Z*_1_ and *Z*_2_ [11]

(19) $$\begin{array}{c} r\left(Z_{i},Z_{j}\right)=\beta \text{do}\left(Z_{j}\right)+\left(1-\beta \right)\text{do}\left(Z_{i}\right). \end{array}$$

Then

(20) $$\begin{array}{c} Z_{i}>Z_{j}\;\text{if}\;\;\text{and}\;\;\text{only}\;\;\text{if}\;\;r\left(Z_{i},Z_{j}\right)>\frac{1}{2}\left(\text{do}\left(Z_{i}\right)+\text{do}\left(Z_{j}\right)\right),\\ Z_{i}<Z_{j}\;\text{if}\;\;\text{and}\;\;\text{only}\;\;\text{if}\;\;r\left(Z_{i},Z_{j}\right)=\frac{1}{2}\left(\text{do}\left(Z_{i}\right)+\text{do}\left(Z_{j}\right)\right) \end{array}$$

and *Z*_*i*_ = *Z*_*j*_ otherwise [11].

The degree of pessimism *β* is submitted by a human being and adjusts ranking of *Z*-numbers to reflect human attitude to the computed do. This attitude may result from the various importance of $\tilde{A}$ and $\tilde{B}$ components of *Z*-numbers for a human being and other issues [11].

## 4. A Method of Decision Making under *Z*-Information Utility Function

Real-world decision relevant information is imprecise, uncertain, and partially reliable. Therefore, results of decision analysis based on such information are also partially reliable. This fact should prevent decision makers relying much on decision analysis results even when a very careful mathematical modeling was used.

A well-known approach to decision making under uncertainty is the use of expected utility function [21]. However, classical paradigm of expected utility function fails to express various adequate decisions due to incapability of handling imperfect decision relevant information. The extension of this paradigm to the framework of *Z*-information may help achieve a more realistic decision analysis technique and, at the same time, use a simple form of the utility function.

Let *𝒮* = {*S*_1_, *S*_2_,…, *S*_*n*_} be a set of states of nature and let *𝒳* = {*X*_1_,…, *X*_*l*_}, $X_{k}=({\tilde{A}}_{k},{\tilde{B}}_{k})$, *k* = 1,…, *l*, be a set of *Z*-valued outcomes. Denote by *ℱ*_*𝒮*_ a *σ*-algebra of subsets of *𝒮*. Then consider *𝒜* = {*f* ∈ *𝒜*∣*f* : *𝒮* → *𝒳*}, the set of *Z*-valued actions, as the set of all *ℱ*_*𝒮*_-measurable *Z*-valued functions from *𝒮* to *𝒳* ^35,32^.

Linguistic information on likelihood *Z*_*P*^*l*^_ of the states of nature is represented by *Z*-valued probabilities $Z_{P_{i}}=({\tilde{A}}_{P_{i}},{\tilde{B}}_{P_{i}})$ of the states *S*_*i*_:

(21) $$\begin{array}{c} Z_{P^{l}}=\frac{Z_{P_{1}}}{S_{1}}+\frac{Z_{P_{2}}}{S_{2}}+\cdots +\frac{Z_{P_{M}}}{S_{M}}. \end{array}$$

In the suggested framework, we extend a classical neo-Bayesian nomenclature as follows: elements of *𝒳* are *Z*-valued outcomes; elements of *𝒜* are *Z*-valued acts; elements of *𝒮* are states of nature; elements of *ℱ*_*𝒮*_ are events.

A framework of decision making with *Z*-information can be formalized as a 4-tuple (*𝒮*, *Z*_*P*^*l*^_, *𝒳*, *𝒜*). The problem of decision making with *Z*-valued information on the basis of EU consists in determination of an optimal act *f* ^*^ ∈ *𝒜*: find *f* ^*^ ∈ *𝒜* for which *Z*_*U*(f^*^)_ ≥ *Z*_*U*(*f*)_, ∀*f* ∈ *𝒜*.

Here *Z*_*U*(*f*)_ is a *Z*-valued expected utility defined as

(22) $$\begin{array}{c} Z_{U(f)}=Z_{X_{1}}Z_{P_{1}}+\cdots +Z_{X_{i}}Z_{P_{i}}+\cdots +Z_{X_{n}}Z_{P_{n}}, \end{array}$$

where multiplication and addition are defined in Section 3.2. The comparison operation ≥ is as defined in Section 3.3.

The suggested approach is based on direct computations with *Z*-numbers, without converting them to fuzzy and/or crisp numbers. This allows preserving available imprecise and partially reliable information and using it in the final comparison of alternatives.

## 5. Practical Applications

In this application section, we intend a problem of decision making in the field of economics. The analyzed data are obtained from Techware Incorporated in [22]. Two new software products were introduced to the market by Techware Incorporated; the company has three alternatives related to these two products: it introduces product 1 only, product 2 only, or both products. The costs for research and development for these two products are $180,000 and $150,000, respectively. The trend of the national economy and the consumers reaction to these products will affect the success of these products in the coming year. If the company introduces product 1, then it will have revenue of $500,000, $260,000, and $120,000 for strong, fair, and weak national economy, respectively. Similarly when product 2 is introduced, there will be revenue of $420,000, $230,000, and 110,000 for strong, fair, and weak national economy, respectively. Finally, when introducing both products 1 and 2, the revenues will be $820,000, $390,000, and $200,000 for strong, fair, and weak national economy, respectively. The experts of the company are very sure that the probabilities of strong and fair economy are about 0.30 and 0.50, respectively. The problem is to determine the best decision.

Let us proceed to formal description of the considered decision problem. The partially reliable linguistic decision relevant information in the considered problem will be described by *Z*-numbers. The set of alternatives is

(23) $$\begin{array}{c} A=\left\{f_{1},f_{2},f_{3}\right\}, \end{array}$$

where *f*_1_ denotes introducing product 1, *f*_2_ denotes introducing product 2, and *f*_3_ denotes introducing both products (1 and 2).

The set of states of nature is

(24) $$\begin{array}{c} S=\left\{S_{1},S_{2},S_{3}\right\}, \end{array}$$

where *S*_1_ denotes strong national economy, *S*_2_ denotes fair national economy, and *S*_3_ denotes weak national economy. The probabilities of states of nature are *Z*_*P*(*S*_1_)_ = (*about*  0.3, *quite* *sure*), *Z*_*P*(*S*_2_)_ = (*about*  0.5, *quite* *sure*).

The set of outcomes is

(25) X=(low,likely),(more  than  low,likely), (medium,likely), (below  compared  to  high,likely), high,likely.

The partially reliable linguistic information for the probabilities of states of nature and the utilities of each alternative taken at different states of nature is shown in Table 1.

The corresponding decision matrix with *Z*-number based representation is shown in Table 2.

The membership functions of the first and the second components of *Z*-numbers for probabilities and utilities from Table 2 are shown in Figures 2–13.

**Figure 13:**
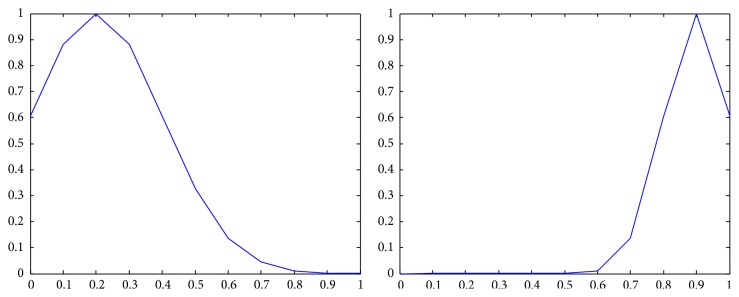
Representation of the first state (*Z*_43_) as a *Z*-number.

Let us proceed to solving the problem. First it is needed to determine unknown *Z*-number based probability *Z*_*P*(*S*_3_)_ = *Z*_43_ = (*A*_43_, *B*_43_) on the basis of *Z*_*P*(*S*_1_)_ = *Z*_41_ and *Z*_*P*(*S*_2_)_ = *Z*_42_. As *Z*_*P*(*S*_3_)_ is completely determined by *Z*_*P*(*S*_1_)_ and *Z*_*P*(*S*_2_)_, its reliability *B*_43_ will be the same as reliabilities *B*_41_ and *B*_42_. Therefore, to complete determination of *Z*_43_ = (*A*_43_, *B*_43_) it is needed to compute *A*_43_ on the basis of *A*_41_ and *A*_42_. For computation of *A*_43_ we used the approach suggested in [19]. The determined *Z*_43_ = (*A*_43_, *B*_43_) is shown in Figure 13.

Based on the previous *Z*-number based data we compute the expected utility for each of the alternatives *f*_1_, *f*_2_, *f*_3_ as follows:

(26) $$\begin{array}{c} Z_{U(f_{1})}=Z_{11}\times Z_{41}+Z_{12}\times Z_{42}+Z_{13}\times Z_{43},\\ Z_{U(f_{2})}=Z_{21}\times Z_{41}+Z_{22}\times Z_{42}+Z_{23}\times Z_{43},\\ Z_{U(f_{3})}=Z_{31}\times Z_{41}+Z_{32}\times Z_{42}+Z_{33}\times Z_{43} \end{array}$$

with multiplication and addition of *Z*-numbers described in Section 3.2.

**Figure 14:**
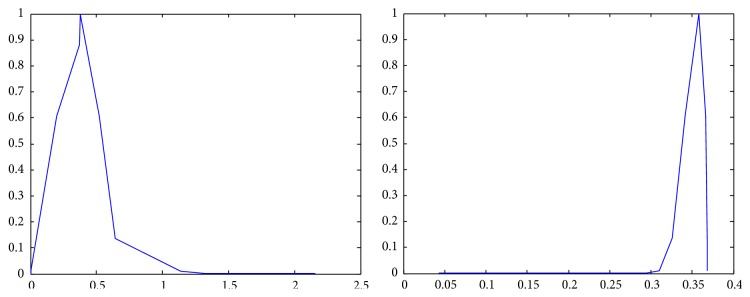
The expected utility results for the first alternative *Z*_*U*(*f*_1_)_.

**Figure 15:**
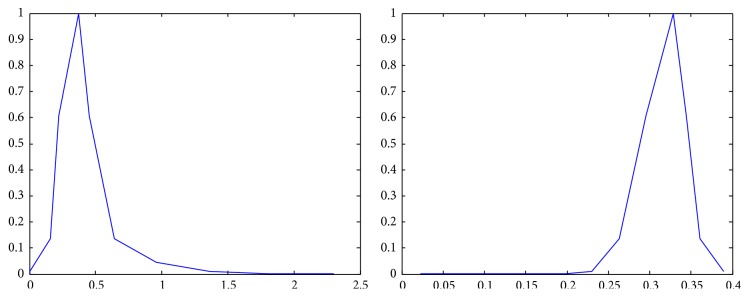
The expected utility results for the second alternative *Z*_*U*(*f*_2_)_.

**Figure 16:**
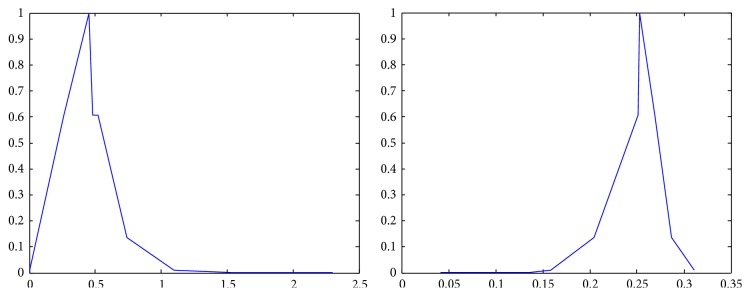
The expected utility results for the third alternative *Z*_*U*(*f*_3_)_.

The results of computation of expected utilities for all the alternatives are shown in Figures 14, 15, and 16.

Now determining the best alternative by comparing the computed *Z*-number valued utilities is needed. For comparison we will use the approach suggested in Section 3.2. In accordance with this principle, at first we obtained the degrees of optimality of the alternatives:

(27) $$\begin{array}{c} \text{do}\left(f_{1}\right)=1,\;\;\text{do}\left(f_{2}\right)=0,\;\;\text{do}\left(f_{3}\right)=0.92. \end{array}$$

As one can see, the second alternative is not Pareto optimal. Now it is needed to compare the first and the third alternatives. Suppose that the pessimism degree in comparison of these alternatives is *β* = 0.3.

Then we have

(28) rZUf1,ZUf3=0.976>12doZUf1+doZUf3=0.96.

Therefore, the best action is *f*_1_.

## 6. Conclusion

The concept of *Z*-numbers opens a door for applications in many areas, especially in decision making theory. The goal of the present study is to develop an approach for decision making under *Z*-information described in NL. The suggested approach utilizes the paradigm expected utility based on a direct computation of *Z*-numbers. The advantage of the approach is its ability to account for imprecision and partial reliability of information and relative simplicity of computations. The approach is applied to solve a benchmark decision problem in the field of economics. The obtained results show validity of the approach.

## Figures and Tables

**Figure 2:**
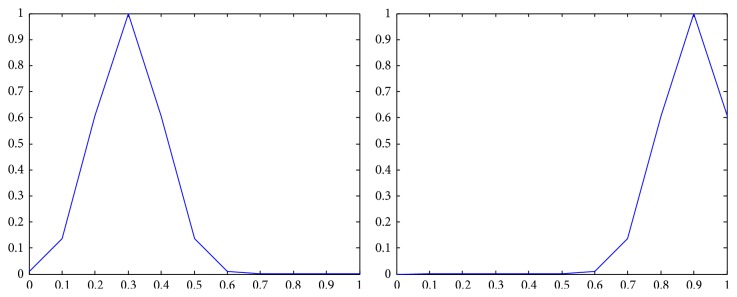
Representation of the first state (*Z*_41_) as a *Z*-number.

**Figure 3 fig3:**
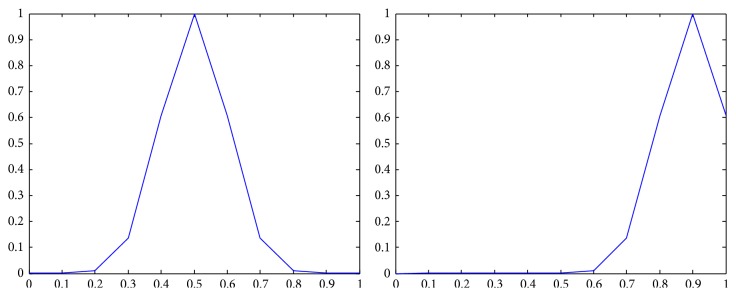
Representation of the second state (*Z*_42_) as a *Z*-number.

**Figure 4 fig4:**
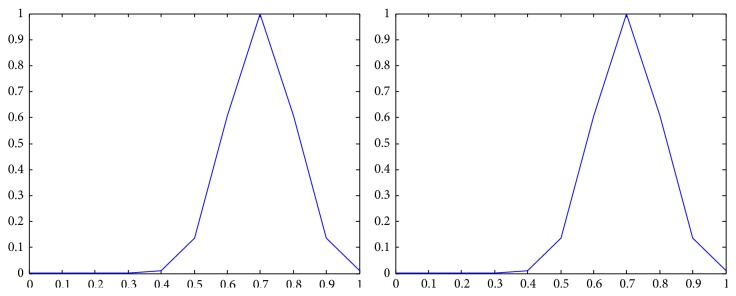
Representation of the first alternative in the first state (*Z*_11_) as a *Z*-number.

**Figure 5 fig5:**
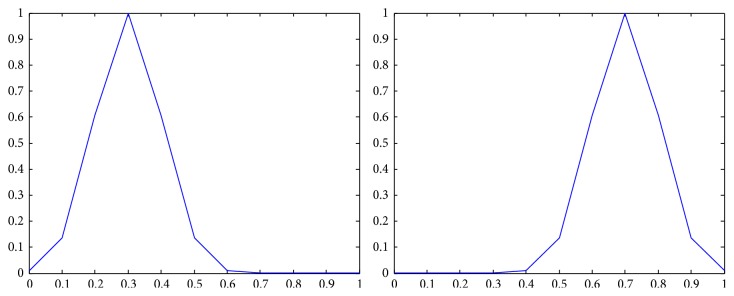
Representation of the first alternative in the second state (*Z*_12_) as a *Z*-number.

**Figure 6 fig6:**
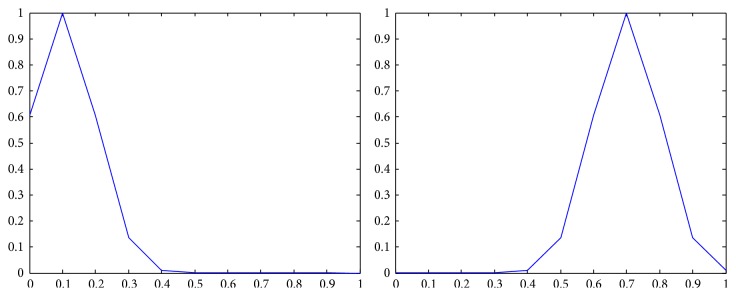
Representation of the first alternative in the third state (*Z*_13_) as a *Z*-number.

**Figure 7 fig7:**
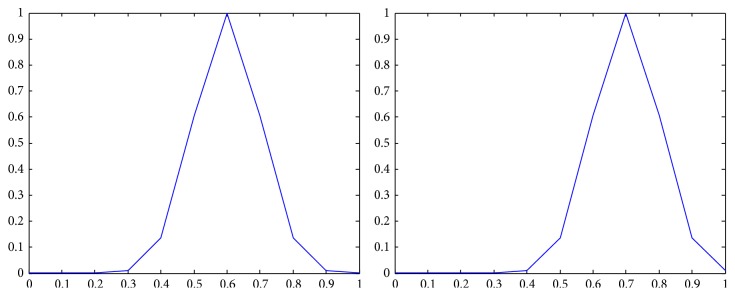
Representation of the second alternative in the first state (*Z*_21_) as a *Z*-number.

**Figure 8 fig8:**
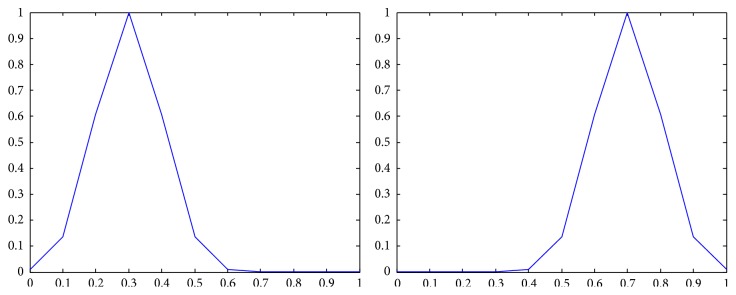
Representation of the second alternative in the second state (*Z*_22_) as a *Z*-number.

**Figure 9 fig9:**
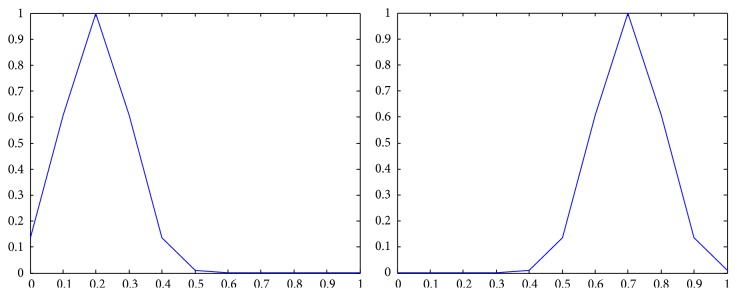
Representation of the second alternative in the third state (*Z*_23_) as a *Z*-number.

**Figure 10 fig10:**
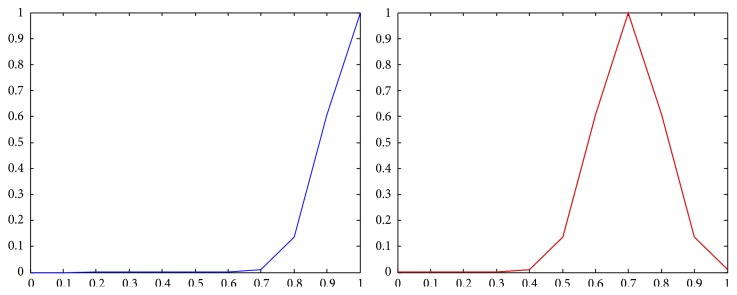
Representation of the third alternative in the first state (*Z*_31_) as a *Z*-number.

**Figure 11 fig11:**
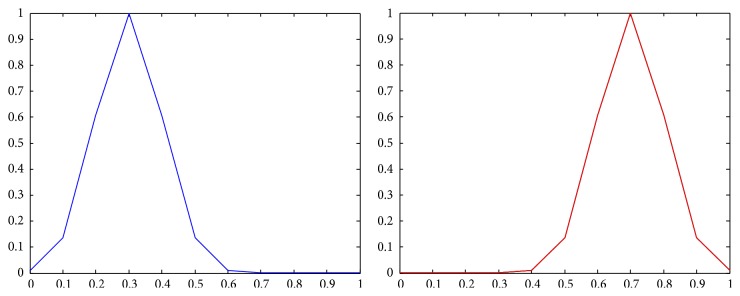
Representation of the third alternative in the second state (*Z*_32_) as a *Z*-number.

**Figure 12 fig12:**
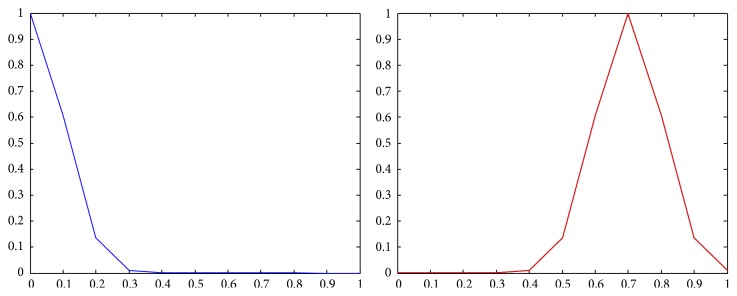
Representation of the third alternative in the third state (*Z*_33_) as a *Z*-number.

**Table 1:** The values of utilities for different alternatives and probabilities of states of nature.

	*S*_1_	*S*_2_	*S*_3_
	(about 0.3, quite sure)	(about 0.5, quite sure)	(about 0.2, quite sure)

*f*_1_	(*High; likely*)	(*Medium; likely*)	(*Low; likely*)
*f*_2_	(*Below compared to high; likely*)	(*Medium; likely*)	(*Low; likely*)
*f*_3_	(*High; likely*)	(*More than low; likely*)	(*Low; likely*)

**Table 2:** Decision matrix with *Z*-number.

	*S*_1_ *Z*_41_	*S*_2_ *Z*_42_	*S*_3_ *Z*_43_

*f*_1_	*Z*_11_	*Z*_12_	*Z*_13_
*f*_2_	*Z*_21_	*Z*_22_	*Z*_23_
*f*_3_	*Z*_31_	*Z*_32_	*Z*_33_
